# Rupture of the Urinary Bladder

**Published:** 1886-06

**Authors:** D. Berrey

**Affiliations:** Secretary West Texas Medical Association, San Antonio


### RUPTURE OF THE BLADDER.

*A. Case by D. Berrey, M. D., Secretary West Texas Medical Association, San Antonio.*

(For Daniel's Texas Medical Journal.)

I WAS called on the morning of the 11th of May, about 10 o'clock, to see Felix Rocha, a Mexican, living on Monteray street, in the suburbs of the city. Was told by the messenger, that on the morning before while on the way to his work on the City and County Hospital, he had an altercation with another Mexican, who kicked him in the abdomen, knocking him down, and kicked him several times while lying on the ground. On arriving at the house I found the patient a man of about 35 years of age, of small stature and slender build. He was in a state of semi collapse, extremeties cold, cold clammy moisture of the skin, and distinct drops of sweat upon the brow. He was sitting upon the side of the bed and expressed himself as unable to lie down on account of the severe pain which the recumbent position produced. The abdomen, externally, presented no sign of violence; there was some bulging in the right hypochondriac region, but otherwise the appearance of the abdomen seemed normal; there was excessive tenderness over the entire abdomen, but it seemed most severe at the point of bulging in the right hypochondria. While I was in the house he passed a quantity of bloody urine, and stated that shortly after receiving the injury his water had been bloody, but later in the day he had several times passed his urine and it was natural in color. He had vomited frequently the day before, and when I saw him he was complaining of constant nausea. I inquired particularly as to the length of time which had elapsed between his passing water and injury, and was told about an hour before he received the kick his bowels and bladder had been evacuated freely. I diagnosed traumatic peritonitis with a lesion of some of the abdominal or pelvic viscera, which particular organ involved it was just then impossible for me to determine. I gave morphia hypodermically, and prescibed one quarter grain of the same drug every two hours; hot applications to be kept constantly applied over the entire abdomen; whiskey to be given frequently, and small quantities of iced milk and egg-nog to be given at stated intervals.

May 11th, 5 p. m. Pain still continues, but not so severe as in the morning; has slept at intervals during the day; pulse about 120 and irregular; temperature, under the tongue, 97°, in the axilla normal. He had passed no urine during the day, so an elastic catheter was introduced and about three ounces of normal colored urine drawn off; ordered the same treatment to be continued.

May 12th, 9 a. m. Patient had slept some during night; had voided a small quantity of urine; temperature normal; pulse about 100, weak and irregular; had vomited a great deal during the night, but the family thought he had retained the greater part of the whiskey, milk and soup which had been given him. Some tympanitis existed, and the bulging in the right hypochondria seemed to be increased; saw no indication for change of treatment, so ordered it continued with the addition of lime-water to the milk.

May 13th, 6 p. m. By request, Dr. C. E. R. King saw the man in consultation. Pretty much the same state of affairs as at my last visit. Tympanitis had somewhat increased; temperature in the axilla normal; pulse about 100 and very irregular; had passed some clear urine during the day.

On consultation, it was determined to substitute opium for morphia, so 1 gr. of opii pulv. with one-eighth gr. of ext. belladonna was ordered every two or three hours; remainder of treatment to be continued.

The probability of rupture of the bladder was discussed, and the propriety of a laparotomy, but the patient's general condition forbade an operation of such magnitude.

May 14th, 10 a, m. Patient decidedly worse than the evening before; extremeties cold, pulse imperceptible, respiration quick and impeded; tympanitis had increased to an alarming extent; abdominal walls exceedingly tense and percussion gave tympanitic resonance over the entire abdomen. On careful palpation we thought we could discover a wave of fluid. It was evident, unless the condition of affairs was relieved, the man could not survive but a short while longer. So, as an exploratory measure, we determined to introduce a aspirating needle in the most prominent part of the swelling. A needle was introduced into the prominence in the right hypochondriac region, when, instead of gas as we expected to find, a dark colored fluid, resembling bloody serum, came bubbling through the instrument. The aspirator failing to work we had to resort to the trocar, which was introduced in the linea alba below the umbilicus, and eleven and one-half pints of a dark-colored fluid, resembling bloody serum, was withdrawn. The fluid was carefully examined, and had none of the odor peculiar to urine about it. A bandage was applied around the abdomen, enemas of milk, brandy and beef peptonoid given, hypodermics of brandy administered, and other treatment ordered continued.

May 14th, 6 p. m. Patient seemed better; respiration free, pulse about 80, and although irregular, was stronger; temperature normal; vomiting still continues.

May 15th, patient vomiting excessively, impossible for him to retain medicine or nourishment, stimulating hypodermics and nutrative enemas ordered every 3 or 4 hours.

May 16th, a. m., patient vomiting bilious looking matter constantly. Hypodermic injections and enemas continued.

May 16th, p. m., patient evidently sinking rapidly; pulse imperceptible at the wrist, extremeties cold and respiration shallow; mental faculties clear. An attempt was still made to save his life, by hypodermic injections of brandy, and the tube of a stomach pump was introduced high up in the bowels, and an enema of a quart of warm milk, 2 ounces of brandy and ½ ounce of beef peptonoid given. In spite of our efforts to sustain the vital powers, the patient gradually sank and died at 9 p. m.

Pursuant to an order issued by Jno. C. Crawford, J. P. acting as coroner, on the 16th day of May at 11 a. m., Dr. C. E. R. King, assisted by Drs. Geo. Cupples and D. Berry, made a post mortem examination on the body of Felix Rocha. The autopsy was made 14 hours after death. Rigor mortis well marked. The adipose tissue of the walls of the abdomen very thin. On laying bare the abdominal cavity some bloody serum escaped, and evidences of general peritonitis were found. The small intestines were inflated with gas, and the large ones contained some fluid fecal matter. The ascending colon was bound down by adhesion. The liver appeared normal in size and color. The gall bladder contained a small quantity of bile; kidneys and spleen healthy. The mucous coat of the stomach was injected, and the organ contained a quantity of brown colored mucus mixed with bilious matter. On examination of the bladder a rupture and contusion, both of considerable size, was found on the anterior surface of the fundus of the organ, incipient gangrene of the abraded surface was also noticed. Death, in our opinion, was due to general peritonitis following rupture of the bladder and extravasation of urine, the result of a blow or blows upon the abdomen.

